# AIDS in men in the city of São Paulo, 1980–2012: spatial and space-time analysis

**DOI:** 10.11606/s1518-8787.2020054001815

**Published:** 2020-11-04

**Authors:** Alessandra Cristina Guedes Pellini, Francisco Chiaravalloti-Neto, Dirce Maria Trevisan Zanetta

**Affiliations:** I Universidade Nove de Julho Faculdade de Medicina São PauloSP Brasil Universidade Nove de Julho. Faculdade de Medicina. Diretoria de Ciências Médicas. São Paulo, SP, Brasil; II Universidade de São Paulo Faculdade de Saúde Pública Departamento de Epidemiologia São PauloSP Brasil Universidade de São Paulo. Faculdade de Saúde Pública. Departamento de Epidemiologia. São Paulo, SP, Brasil

**Keywords:** Acquired Immunodeficiency Syndrome, epidemiology, Space-Time Analysis, Spatial analysis, Morbidity and mortality indicators, Ecological studies

## Abstract

**OBJECTIVES:**

To identify spatial and space-time clusters with high incidence rates of AIDS in men living in the city of São Paulo since the first case of the disease in 1980.

**METHODS:**

HIV/AIDS notifications were obtained from the Notifiable Diseases Information System (57,440 men) between January 1980 and June 2012. The cases were geocoded by residence address; then analyses of purely spatial, space-time and spatial variation in temporal trends were performed for three sets of data: total cases of AIDS in men aged 13 years or older, men aged 50 years or older, and deaths from AIDS.

**RESULTS:**

It was possible to geocode a significant proportion of AIDS cases (93.7%). In the purely spatial scanning analysis, considering the entire period evaluated, the AIDS epidemic in men presented an important spatial concentration in the Center and in contiguous areas of the North, Southeast and West regions of the municipality, regardless of age group and evolution to death (relative risks between 1.22 and 5.90). Considering space and time simultaneously, several clusters were found, spread throughout all regions of the municipality (relative risks between 1.44 and 8.61). In the analysis of spatial variation in temporal trends, the clusters in the most peripheral regions presented a higher annual percentage increase in disease rates (up to 7.58%), denoting the tendency of “peripherization” of the epidemic in men in the city of São Paulo.

**CONCLUSIONS:**

This study allowed the detection of geographic clusters of high risk for AIDS in men, pointing to priority areas in the municipality, both for programmatic actions and to guide other studies.

## INTRODUCTION

In the early 1980s, the epidemic of the human immunodeficiency virus/ acquired immunodeficiency syndrome (HIV/AIDS) in Brazil mainly affected the metropolitan regions of São Paulo and Rio de Janeiro^1^^–^^4^. The cases were mostly homosexual or bisexual men, with a high socioeconomic level, in addition to patients with hemophilia and recipients of blood transfusions^4^^,^^5^. The disease evolved unevenly in the country, with many transformations in its epidemiological profile.

The analysis of the geographical heterogeneity of HIV/AIDS morbidity and mortality is fundamental for the surveillance and monitoring of trends of the disease. However, investigating the spatial structure of this epidemic is still a challenge, because large geographical areas with low population density can mask spatial variation and lead to a misinterpretation of the true underlying geographic patterns^6^. Ecological studies can provide a more consistent response to the presence of an association than studies based on individuals, especially when the variability of an exposure within each unit of analysis is limited^7^.

Some statistical methods help detect relevant disease clusters due to actual effects and not chance, helping in the allocation of resources^8^. Several approaches to HIV/AIDS using spatial and space-time scanning analysis techniques have been explored by different authors, in studies conducted in African countries^6^^,^^9^^–^^11^, as well as in the United States^12^^,^^13^, Canada^14^^,^, China^15^ and Brazil^1^^,^^4^^,^^5^^,^^16^^–^^19^.

HIV/AIDS has already completed almost 40 years of history in the city of São Paulo; however, little was studied about space as a determinant of disease trends in this location. The objective of this study was to identify spatial and space-time clusters with high incidence rates of AIDS in men (considered here regarding biological sex), living in the city of São Paulo, since the first case of the disease, identified in 1980.

## METHODS

### Study Design and Location

This study has a descriptive ecological design and was developed in the city of São Paulo, with an estimated population of 11.8 million inhabitants in 2019, 47.6% of them men. The city's area is 1,521.11 km² and the population density is 7,765.06 inhabitants/km², according to the State Data Analysis System Foundation^20^. The geographic coordinates of the central point of the municipality of São Paulo are latitude of 23°32′51″ South and longitude of 46°38′10″ West.

**Figure 1:**
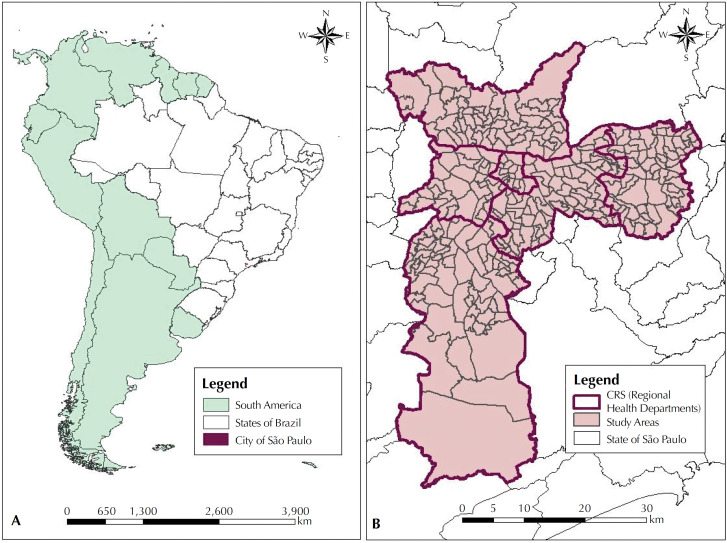
(A) Map of Brazil, according to federated states, with emphasis on the city of São Paulo. (B) Map of the municipality of São Paulo, according to CRS and study areas.

Source: Digital maps of South America and Regional Health Departments: CEInfo - São Paulo Municipal Health Department (2018). Digital maps of Brazil, federated states, city of São Paulo and study areas: Brazilian Institute of Geography and Statistics (2018).

For the planning of healthcare actions, the municipality is divided into six *Coordenadorias Regionais de Saúde* (CRS – Regional Health Departments): North, Center, West, Southeast, East and South. The study includes 310 specifically chosen key study areas (Figure 1), formed by mutually exclusive agglomerates of census tracts of the Brazilian Institute of Geography and Statistics (IBGE)^21^.

### Data Sources

The data for this study come from the HIV/AIDS notification database of the *Sistema de Informação de Agravos de Notificação* (SINAN – Notifiable Diseases Information System), from the Health Surveillance Coordination of the city of São Paulo. The cartographic base was obtained from the Center for Metropolitan Studies (CMS)^22^. The digital meshes and populations of the areas were acquired on the IBGE website^21^.

### Population and Study Period

We chose to describe the trajectory of AIDS exclusively in males, since, in the city of São Paulo, among the 88,424 cases reported between 1980 and 2013, 72.2% were men^23^, a higher proportion than those in the state of São Paulo (68.9%)^24^ and Brazil (64.9%)^25^. A total of 57,440 cases of AIDS in men living in the city of São Paulo were registered in SINAN during the study period, from January 1980 to June 2012.

### Geocoding of AIDS Cases

The geocoding of AIDS cases was performed according to the home address (street, number, complement, reference, neighborhood and ZIP Code), for the comparison with the CMS-produced digital map of the city of São Paulo^22^ of 2014, available for free on the Internet, using the Software MapInfo Professional (version 11.0), in addition to complementary systems based on Google Earth.

It was necessary to standardize the addresses, due to many inaccuracies and lack of uniformity, as well as to make them compatible with the digital street database, before obtaining the geographical locations. Quality of geocoding was verified by checking 10% of the geocoded addresses, randomly drawn, and overlapping the layer of the geocoded points to the layer of addresses of the city, obtaining 96% accuracy.

### Statistical Scan Analysis

This study aimed to detect significantly high geographic clusters of AIDS in men in space and space-time. The statistical scanning analyses were performed in the SaTScan^TM^ program (version 9.4.2)^26^, assuming that the number of cases in each area follows Poisson distribution, according to a known under-risk population^6^^,^^8^^,^^14^^,^^26^. The maximum population size for the scanning window was 5%. The p-value of the clusters was obtained through the Monte Carlo hypothesis test, with 999 replications (SaTScan^TM^ standard)^26^. For more details on the theory behind spatial and space-time scanning statistics, consultation on the Kulldorff references is recommended^8^^,^^26^^,^^27^.

The trajectory of AIDS cases was analyzed by three modalities of scanning statistics: (1) purely spatial; (2) space-time; (3) spatial variation in temporal trends. Scanning statistics are used to detect and evaluate clusters of cases in a purely temporal, purely spatial, or space-time configuration. This is done through the gradual scanning of a window over time and/or space, indicating the expected and actual number of observations inside the window in each location^26^.

Maps with significant clusters (p < 0.05) and respective relative risks (spatial and space-time scanning analyses) as well as internal and external temporal trends (spatial variation in temporal trends analysis) were elaborated in the ArcGIS application (version 10.1).

For each modality, three sets of data were evaluated: (a) all cases of AIDS in men aged 13 years or older – the age group considered by surveillance as “AIDS in adults”^28^; (b) men aged 50 years or older, due to the increasing importance of AIDS cases at older ages, observed since the late 1990s^2^^,^^3^; (c) AIDS deaths in men aged 13 or older. For each analysis, it was necessary to create three files, as required by SaTScan^TM26^:

1. Case file: Contains the number of cases in each area. For the space-time analyses, the time reference was the date of AIDS diagnosis, in the year/month format. Age was included as a covariate, stratified in the following age groups: 10–19; 20–29, 30–39, 40–49, 50–59, 60–69, and 70 or more.
2. Population archive: comprises a population size at risk for each combination of locality, time (in years) and covariate. Populations according to age groups were specified for each area in census years (1980, 1991, 2000 and 2010). For the years between each census, the SaTScan^TM^ performed linear interpolation based on the populations of the censuses immediately before and after^26^.
3. Spatial coordinate archive: pairs of latitude and longitude coordinates of the centroids of the study areas, used as a proxy for the location of the study participants^6^^,^^11^.

### Ethical Aspects

The use of the data for the purposes of this study was approved by the Research Ethics Committee of the School of Public Health of the University of São Paulo (opinion embodied no. 261,202, of April 19, 2013). For any circumstances, the norms and guidelines regulating research involving human beings were considered (resolution no. 466 of December 12, 2012, of the National Health Council).

## RESULTS

Among the 57,440 cases of AIDS in men, 53,825 were geolocated in several stages, reaching a final geocoding rate of 93.7%. Fifty-three cases with ignored age were excluded. Thus, for each data set, the following were analyzed: (a) 53,772 cases of men aged 13 years or older; (b) 5,272 cases of men aged 50 years or older; (c) 28,981 deaths caused by AIDS.

**Figure 2:**
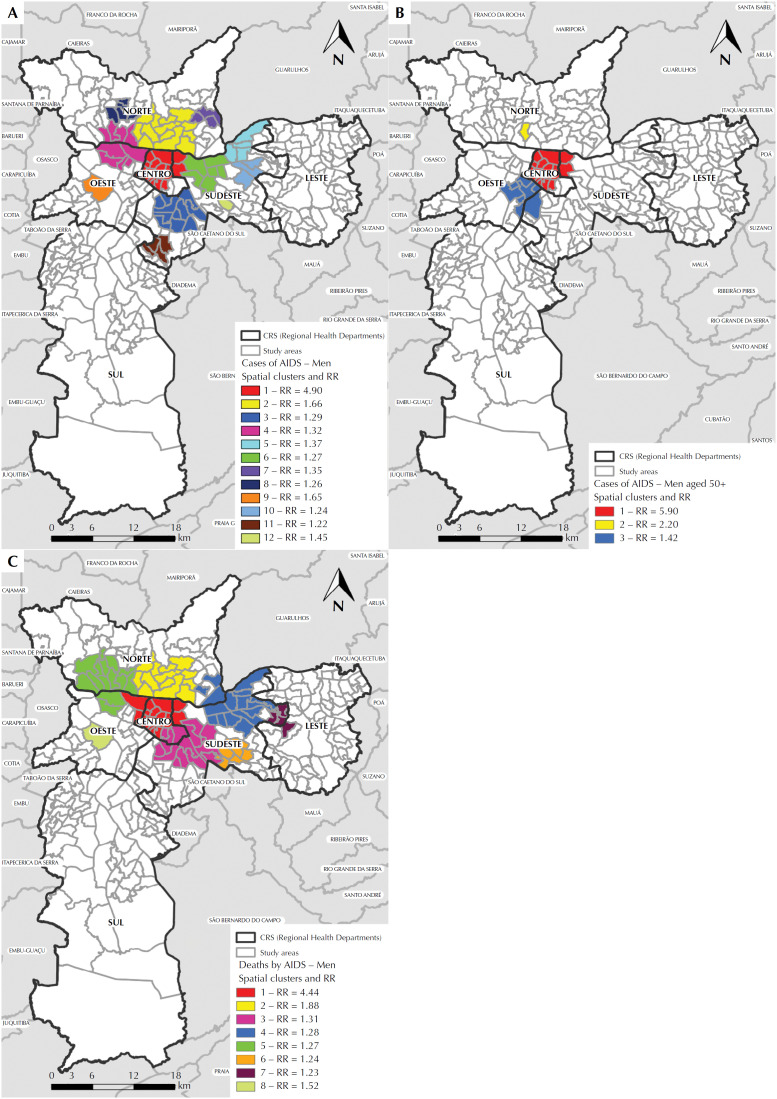
Maps of purely spatial clusters with high rates of AIDS cases in men, according to study area, Regional Health Department (CRS) and relative risk (RR): (A) total cases in men aged 13 years or older; (B) total cases in men aged 50 years or older; (C) deaths of men aged 13 years or older. City of São Paulo, 1980 to June 2012.

Source: AIDS cases: Notifiable Diseases Information System - Center for Disease Control of the Health Surveillance Coordination of the Municipal Health Department of São Paulo. Population: Datasus/Brazilian Institute of Geography and Statistics (IBGE). Study areas: IBGE census (2010). CRS: CEInfo - São Paulo Municipal Health Department.

Regarding the purely spatial analysis of the total cases, 12 statistically significant high-risk spatial clusters (p < 0.05) were identified. Throughout the analyzed period, the epidemic was concentrated in the central area, with the main cluster with relative risk (RR) of 4.90 (Figure 2A). The two secondary clusters of greater statistical significance were located, respectively, to the North and Southeast of the municipality, close to the main cluster (Figure 2A).

In the analysis of cases aged 50 years or older, three significant clusters were identified. The main one, with RR of 5.90 (Figure 2B) occupied the same central area of the main cluster of all cases (Figure 2A).

In the spatial scanning analysis of deaths, eight significant clusters were found. The main one, with RR of 4.44 (Figure 2C) remained in the central region, involving the same previous area, in addition to two new ones to the West (Figure 2C). In the purely spatial scanning analysis, considering the entire period evaluated, the AIDS epidemic in men presented an important spatial concentration in the Center and in contiguous areas of the North, Southeast and West regions of the municipality, regardless of age group and evolution to death.

**Figure 3:**
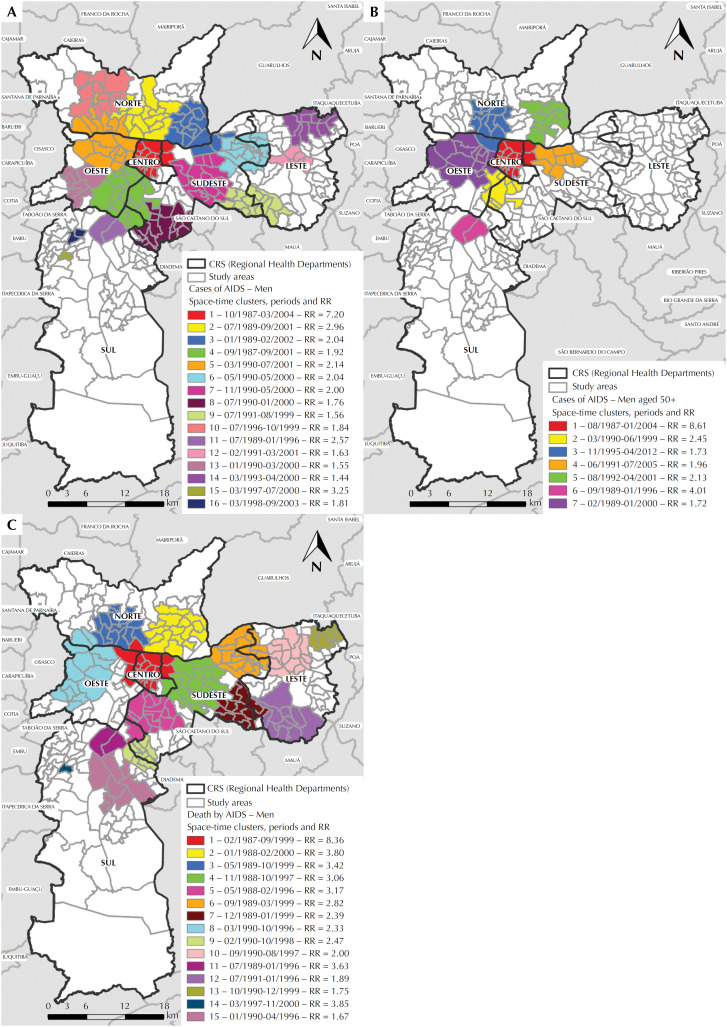
Maps of the space-time clusters with high rates of AIDS cases in men, according to the study area, Regional Health Department (CRS), cluster period and relative risk (RR): (A) total cases in men aged 13 years or older; (B) total cases in men aged 50 years or older; (C) deaths of men aged 13 years or older. City of São Paulo, 1980 to June 2012.

Source: AIDS cases: Notifiable Diseases Information System - Center for Disease Control of the Health Surveillance Coordination of the Municipal Health Department of São Paulo. Population: Datasus/ Brazilian Institute of Geography and Statistics (IBGE). Study areas: IBGE census (2010). CRS: CEInfo - São Paulo Municipal Health Department.

Through the space-time scanning statistics of the total cases, 16 significant clusters were identified. The main one, with RR of 7.20 (Figure 3A) occurred between October 1987 and March 2004, in the Center region. The other 15 secondary clusters, mainly concentrated from 1993 to 1999, were distributed in the other regions of the municipality, including the CRS South, which had three clusters (Figure 3A).

In the age group aged 50 years or more, seven significant clusters were detected. The main one, with RR of 8.61 (Figure 3B), was located in the same central area of the main clusters in purely spatial analyses, and five other secondary clusters occurred in the Southeast, West and North, bordering the main one. The most critical periods occurred between 1992 and 1999; however, we highlight a cluster with RR of 1.73 that lasted until 2012, the most recent year of the study (Figure 3B).

In the space-time analysis of deaths, 15 significant clusters were found. The main one, with RR of 8.36 (Figure 3C) remained in the central region, now wider, between February 1987 and September 1999. We highlight the expansion of secondary clusters in the Eastern CRS, in addition to three clusters located exclusively in the South region. The most critical period occurred between 1990 and 1996 (Figure 3C). In the scanning analysis that considered space and time simultaneously, several clusters were found scattered throughout all regions of the municipality.

**Figure 4:**
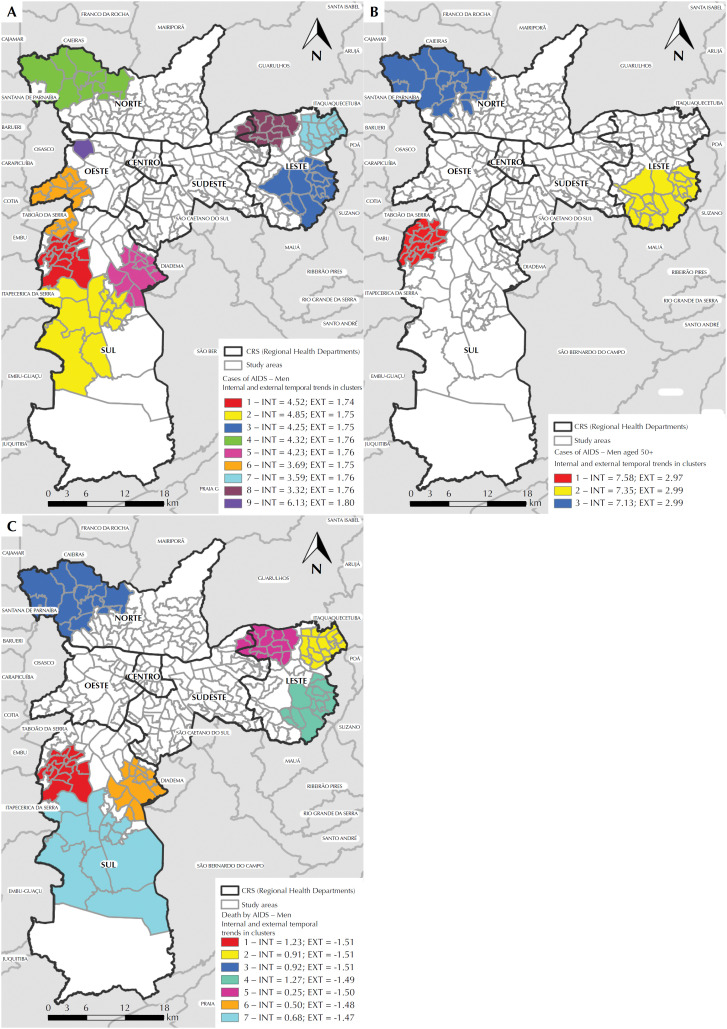
Maps of clusters of spatial variation in temporal trends with high trends of AIDS cases in men, according to the study area, Regional Health Department (CRS), temporal trend of annual increment (%) inside (INT) and outside (EXT) of the cluster: (A) total cases in men aged 13 years or older; (B) total cases in men aged 50 years or older; (C) deaths of men aged 13 years or older. City of São Paulo, 1980 to June 2012.

Source: AIDS cases: Notifiable Diseases Information System - Center for Disease Control of the Health Surveillance Coordination of the Municipal Health Department of São Paulo. Population: Datasus/ Brazilian Institute of Geography and Statistics (IBGE). Study areas: IBGE census (2010). CRS: CEInfo - São Paulo Municipal Health Department.

In the analysis of spatial variation in temporal trends, nine significant clusters were found in the total set of cases. The values of internal temporal trends (INT) and external (EXT) of the clusters are presented. With an internal annual increase of 4.52%, the most significant cluster occurred in the Southern region of the city (Figure 4A).

In the age group aged 50 years or more, three significant clusters were detected. The main one, with an internal annual increase of 7.58%, was also located in the South CRS, near the border with the West region (Figure 4B).

In the subset of deaths, seven significant clusters were detected. The main one, with an internal annual increase of 1.23% (Figure 4C), involved the same areas and was in the same region of the main cluster of all cases (Figure 4A).

Regardless of the age group and the evolution of AIDS cases in men, there are spatial clusters, especially in the most peripheral regions of the city, which showed a higher annual percentage increase in the rates of the disease within the clusters, compared to the areas outside them, which denotes a tendency to “peripherize” the epidemic in men in this municipality.

The table summarizes the main characteristics of the statistically significant main and secondary clusters of AIDS cases in men in the three data sets analyzed, identified in the scan analyses of purely spatial, space-time and spatial variation in temporal trends.

**Table:** Characteristics of the main and secondary clusters statistically significant (p < 0.05) of high AIDS rates in men, identified in the statistical analysis of purely spatial, space-time and spatial variation in temporal trends, according to different data sets analyzed. City of São Paulo, 1980 to June 2012.

Dataset	Purely spatial scanning analysis								
	Type of cluster	n clusters	n localities	n cases observed	n cases expected	Obs/Exp^a^	relative risk		p
Men – 13 years of age or older	Main	1	15	9,164	2,163.1	4.24	4.90		< 0.0001
	Secondary^b^	11	61	14,175	10,358.3	1.21–1.64	1.22–1.66		< 0.0021
Men – 50 years of age or older	Main	1	15	1,016	204.9	4.96	5.90		< 0.0001
	Secondary^b^	2	9	220	145.7	1.40–2.19	1.42–2.20		< 0.013
Deaths in men	Main	1	16	4,603	1,181.1	3.90	4.44		< 0.0001
	Secondary^b^	7	69	8,667	6,276.7	1.23–1.81	1.23–1.88		< 0.036

Dataset	Space-time scanning analysis								
	Type of cluster	n clusters	n localities	n cases observed	n cases expected	Obs/Exp^a^	relative risk	Period^c^	p
Men – 13 years of age or older	Main	1	15	6,880	1,074.1	6.40	7.20	10/1987-03/2004	< 0.0001
	Secondary^b^	15	163	15,645	7,916.9	1.44–3.24	1.44–3.25	09/1987-09/2003	< 0.026
Men – 50 years of age or older	Main	1	15	729	96.5	7.56	8.61	08/1987-01/2004	< 0.0001
	Secondary^b^	6	55	715	367.2	1.70–3.99	1.72–4.01	02/1989-04/2012	< 0.05
Deaths in men	Main	1	19	3,904	529.8	7.37	8.36	02/1987-09/1999	< 0.0001
	Secondary^b^	14	156	9,991	3,653.7	1.66–3.85	1.67–3.85	01/1988 –11/2000	< 0.0001

Dataset	Spatial variation in temporal trends analysis								
	Type of cluster	n clusters	n localities	n cases observed	n cases expected	Obs/Exp^a^	relative risk	Trend within the cluster	p
Men – 13 years of age or older	Main	1	14	1,571	2,648.3	0.59	0.58	4.52	0.001
	Secondary^b^	8	95	10,580	17,144.8	0.46–0.79	0.45–0.78	3.32–6.13	< 0.0041
Men – 50 years of age or older	Main	1	17	179	259.7	0.69	0.68	7.58	0.003
	Secondary^b^	2	34	337	485.0	0.66–0.73	0.65–0.72	7.13–7.35	< 0.03
Deaths in men	Main	1	14	790	1,424.6	0.55	0.54	1.23	0.001
	Secondary^b^	6	80	4,488	7,731.8	0.40–0.78	0.39–0.77	0.25–1.27	< 0.011

a Obs/exp: relationship between observed and expected cases.

b The characteristics of the secondary clusters were summarized by the sum of the number of localities, observed cases and expected cases of all clusters. The ratio between observed and expected, relative risks and internal trends of clusters are presented according to minimum and maximum values.

c The period reported for secondary clusters refers to the first date (month/year) of the earliest identified space-time cluster, and the final date (month/year) of the cluster that had the later end.

Source: SaTScan^TM^ output information file (version 9.4.2, 2015).

## DISCUSSION

This study sought to identify spatial and space-time clusters of AIDS in men aged 13 years or older in the city of São Paulo, from the first case in 1980 to June 2012. Several methods analyze global and local clusters, with differences in performance in terms of power and precision^4^^–^^6^^,^^9^^–^^19^. In this study, the scanning method was chosen, with the objective of identifying locations with high concentration of AIDS in men.

The progression of HIV in populations does not occur randomly and uniformly, but rather due to intimate and complex social behaviors among two or more people^11^^,^^14^. Despite the determining role of such interactive behaviors in the transmission of the disease, the individual-based approach is not sufficient for a full evaluation of the role played by the interactions of the groups, which result in the dissemination of HIV^14^. For the transmission of the disease to occur, many small groups must be linked to each other, or even to larger components, by unidentified connections. The information of these small components can be difficult to analyze, given the lack of knowledge related to other components to which they may be linked^14^.

The use of spatial analysis tools allows identifying differentials in HIV/AIDS behavior in populations and in the spaces they occupy. Such techniques also contribute to the identification of locations where there is a higher risk of falling ill or dying due to the disease, which can help select areas for specific policies and interventions by the competent agencies^29^.

The purely spatial scanning analysis showed that AIDS was concentrated in the Central region of the city of São Paulo, regardless of age group and evolution to death. The 2014 São Paulo City Epidemiological Bulletin on AIDS, HIV and STD^30^ mentions an epidemic concentrated in the central region, despite the significant reduction in the incidence rate observed in this area between 2005 and 2013. A study conducted in 2015 identified an HIV prevalence of 15.4% among men who have sex with men (MSM) at the Central region, with more than half of the population studied not knowing about their seropositivity, raising questions about a “new wave” of the disease epidemic^31^.

The relative risk of the cluster of men aged 50 years or older was the highest among all those observed in purely spatial analyses. Between 1996 and 2005, the incidence rate of AIDS among men aged 50 to 59 years increased from 18.2 to 29.8 cases/100,000 men. In those over 60 years, in the same period, there was also an increase in the rate, from 5.9 to 8.8. Until 2010, this increase remained in the age groups above 50 years^2^^,^^3^.

The “aging” of the AIDS-infected population has been highlighted as a trend of the epidemic in Brazil, and the older population are rarely the target of interventions to reduce contagion^2^^,^^15^^,^^32^. Considering the natural history of the disease, it is valid to assume that HIV infection in this group occurred at least 10 years before the manifestation of AIDS. It is inferred that the older population started their sexual life when HIV/AIDS did not yet exist, so that they are less likely to use condoms, presenting greater exposure to the virus. Some authors also consider that the increased incidence in older men may reflect the use of drugs for erectile dysfunction, a contributing factor for active sex life in the older population^1^^,^^32^.

According to recent data from the Ministry of Health, in the last decade there has been an increase in the rate of AIDS detection in men aged 15 to 29 years and aged 55 years or more, especially for young people aged 15 to 24 years^25^. The Research of Knowledge, Attitudes and Practices in the Brazilian Population^33^, conducted in 2013 with individuals aged 15 to 64 years, pointed out that, among the reasons for the “second wave” of infection between MSM, are the low rate of testing (86.1% of males had never been tested for HIV) and the occurrence of a higher number of casual partnerships among young people, in addition to a higher percentage of sexual relations with partners met via Internet^33^.

In the scan analysis of space-time, an expressive number of clusters were observed, denoting that all regions of the municipality were affected at some point, except the South. The main cluster remained between October 1987 and March 2004, in the city center, including 1992, which had the highest incidence rate of AIDS in adult men (88.1 cases/100,000 men)^29^.

The similarity of the subset of deaths with the one that included all cases can be explained by the high lethality of the disease in men (54.7%), especially between 1990 and 1996^29^. Since that year, advances in the treatment of the disease, such as the free distribution of highly active antiretroviral therapy (HAART) by the Ministry of Health, have resulted in a significant improvement in patient survival, with a drop in mortality by about 50% and a reduction in opportunistic infections^16^.

The peak of AIDS mortality in men in the city of São Paulo occurred in 1994 (52.5/100,000 men)^23^^,^^30^; since then, measures aimed at care resulted in a significant decrease in the crude death rate (CDR), which in 2012 was 10.4/100,000 men^23^. In the same year, in the state of São Paulo, this rate was 9.1/100,000 men^24^, and in Brazil, 8.3/100,000 men^25^.

An analysis conducted with the death certificates of the Mortality Information System (MIS) with mention of HIV/AIDS in any field of completion of causes of death showed that in Brazil, between 2000 and 2007, there was an important increase in the percentage of deaths of people infected with the virus due to basic causes not related to HIV/AIDS, from 2.5% to 7.0%. This fact indicates that other diseases (malignant neoplasms, cardiovascular diseases, diseases of the digestive and respiratory tract, metabolic and endocrine diseases, viral hepatitis and external causes) have increasingly impacted the survival of people with HIV/AIDS^2^.

Factors such as late diagnosis, co-infections, lack of access to health resources, among others, affect people living with HIV/AIDS more intensely in some locations than in others^34^. Early diagnosis of HIV, in order to ensure timely treatment, adherence to treatment, strengthening of the genotyping network and the inclusion of new drugs are fundamental strategies to reduce AIDS mortality and improve the quality of life of people living with the virus^29^^,^^34^.

In this study of the trajectory of AIDS in the city of São Paulo, a tendency of “peripherization” of the disease was evidenced in men, since more peripheral areas presented, over time, a greater percentage increase in rates within the clusters than the areas outside them. It is noteworthy that a statistically significant cluster in this type of analysis does not necessarily imply that the overall rate of disease is higher or lower in it, since the scan statistic of spatial variation in temporal trends is not looking for clusters of high or low risk, but rather seeking clusters with a higher or lower internal trend than the trend outside them^26^.

The poorest population is the most affected by AIDS, due to several aspects related to vulnerability, such as lack of resources for HIV prevention; difficulty in accessing health services; embarrassment, pressure and threats; precariousness of housing, food and employment; low schooling and difficult access to information^35^. The presence of the AIDS epidemic in contexts of social vulnerability aggravates the already precarious conditions of these people, as undesirable living conditions accumulate^29^.

In 1994, the “pauperization” of AIDS was already indicated, which mainly affected the poorest social class and residents of the peripheral regions^36^. The process of “pauperization” represented one of the trends of the epidemic of the disease in Brazil, which increasingly affected people with a lower level of education and users of injectable drugs^4^^,^^5^^,^^16^^,^^36^. Territorial exclusion is related to social exclusion, reflecting the processes of urbanization and urban segregation^37^. The center and peripheral zones of the city of São Paulo represent an example of this inequality.

This study presents some limitations, among which it is noteworthy that only cases of AIDS were studied, not including those of asymptomatic HIV, because only AIDS was a mandatory notification condition during the study period. The prolonged latency period of AIDS makes it difficult to characterize the place where individuals acquired HIV, because when they are reported with AIDS-defining infections, a long time has passed since the moment of infection. Other limitations relate to the address of patients informed in the AIDS notification and investigation forms, which may not represent the actual place of residence, because inaccurate addresses are provided in order to gain access to the care network outside the region of residence.

It is also considered the possibility of underreporting of the disease in surveillance and mortality systems, especially in the first decades of the epidemic, due to changes in the versions of the International Classification of Diseases (in the ICD-9, in force until 1995, there was no specific category for AIDS), besides the stigma related to the disease, which may have contributed to the non-mention of this cause in death certificates. In addition, the scanning techniques do not allow to verify associations between exposure and outcome, revealing only the localities with higher risk or protection in the rates of the disease.

The study strength is the highlighting of the significant proportion of geocoded AIDS cases (93.7%). The ecological design allowed to evidence effects not detected at the individual level, and the choice of strictly spatial, space-time and spatial variation in temporal trends techniques allowed the detection of geographic clusters of high risk of AIDS in men.

In conclusion, the AIDS epidemic in men was concentrated in the central region of the city of São Paulo and in contiguous areas of the North, Southeast and West regions, regardless of the age group analyzed and the evolution to death. Over time, the disease reached men in all regions of the municipality, with a tendency to “peripherization.” The study helped to reveal priority areas in the city of São Paulo, both for programmatic actions in the scope of HIV/AIDS and to guide other studies.
