# Supplementary material for: AIDS in men in the city of São Paulo, 1980–2012: spatial and space-time analysis
Source: Rev Saude Publica. 2020 Nov 4;54:96. doi: 10.11606/s1518-8787.2020054001815 (PMC7647470; doi:10.11606/s1518-8787.2020054001815)
Supplement: Translation

# Translation: Aids em homens no município de São Paulo, 1980–2012: análise espacial e espaço-temporal

**Authors:** Alessandra Cristina Guedes Pellini, Francisco Chiaravalloti-Neto, Dirce Maria Trevisan Zanetta

**Affiliations:** I São PauloSP Brasil Universidade Nove de Julho. Faculdade de Medicina. Diretoria de Ciências Médicas. São Paulo, SP, Brasil; II São PauloSP Brasil Universidade de São Paulo. Faculdade de Saúde Pública. Departamento de Epidemiologia. São Paulo, SP, Brasil

**Keywords:** Síndrome de Imunodeficiência Adquirida, epidemiologia, Análise Espaço-Temporal, Análise Espacial, Indicadores de Morbimortalidade, Estudos Ecológicos

## Abstract

**OBJETIVOS:**

Identificar aglomerados espaciais e espaço-temporais de altas taxas de incidência de aids em homens residentes no município de São Paulo desde o primeiro caso da doença em 1980.

**MÉTODOS:**

As notificações de HIV/aids foram obtidas do Sistema de Informação de Agravos de Notificação (57.440 homens) entre janeiro de 1980 e junho de 2012. Os casos foram geocodificados por endereço de residência; em seguida, análises de varredura puramente espacial, espaço-temporal e de variação espacial nas tendências temporais foram realizadas para três conjuntos de dados: total de casos de aids em homens com 13 anos de idade ou mais, homens com 50 anos ou mais e óbitos por aids.

**RESULTADOS:**

Foi possível geocodificar uma expressiva proporção de casos de aids (93,7%). Na análise de varredura puramente espacial, considerando-se todo o período avaliado, a epidemia de aids nos homens apresentou importante concentração espacial no Centro e em áreas contíguas das regiões Norte, Sudeste e Oeste do município, independentemente da faixa etária e da evolução para o óbito (riscos relativos entre 1,22 e 5,90). Levando-se em conta simultaneamente o espaço e o tempo, diversos aglomerados foram encontrados, espalhados por todas as regiões do município (riscos relativos entre 1,44 e 8,61). Na análise da variação espacial nas tendências temporais, os aglomerados nas regiões mais periféricas apresentaram maior incremento percentual anual das taxas da doença (de até 7,58%), denotando a tendência de “periferização” da epidemia nos homens na cidade de São Paulo.

**CONCLUSÕES:**

Este estudo permitiu a detecção de aglomerados geográficos de alto risco para a aids nos homens, apontando para áreas prioritárias no município, tanto para ações programáticas como para nortear outros estudos.

## INTRODUÇÃO

No início da década de 1980, a epidemia do vírus da imunodeficiência humana/síndrome da imunodeficiência adquirida (HIV/aids) no Brasil atingia principalmente as regiões metropolitanas de São Paulo e Rio de Janeiro^1^^–^^4^. Os casos eram, em sua maioria, homens homossexuais ou bissexuais, com alto nível socioeconômico, além de portadores de hemofilia e receptores de sangue^4^^,^^5^. A doença evoluiu desigualmente no país, com muitas transformações em seu perfil epidemiológico.

A análise da heterogeneidade geográfica da morbimortalidade do HIV/aids é fundamental para a vigilância e o acompanhamento das tendências desse agravo. Contudo, investigar a estrutura espacial dessa epidemia ainda é um desafio, pois grandes áreas geográficas com baixa densidade populacional podem mascarar a variação espacial e levar a uma má interpretação dos verdadeiros padrões geográficos subjacentes^6^. Estudos ecológicos podem fornecer uma resposta mais consistente em relação à presença de uma associação do que os estudos baseados em indivíduos, sobretudo quando a variabilidade de uma exposição dentro de cada unidade de análise é limitada^7^.

Alguns métodos estatísticos auxiliam a detectar aglomerados de casos de doenças não devidos ao acaso, ajudando na alocação de recursos^8^. Diversas abordagens do HIV/aids utilizando técnicas de análise de varredura espacial e espaço-temporal já foram exploradas por diferentes autores, em estudos realizados na África^6^^,^^9^^–^^11^, nos Estados Unidos^12^^,^^13^, no Canadá^14^, na China^15^ e no Brasil^1^^,^^4^^,^^5^^,^^16^^–^^19^.

O HIV/aids já completa quase 40 anos de história no município de São Paulo (MSP); contudo, pouco se estudou a respeito do espaço como determinante das tendências da doença nesse local. O objetivo deste trabalho foi identificar aglomerados espaciais e espaço-temporais de altas taxas de incidência de aids em homens (considerados aqui em função do sexo biológico), residentes no município de São Paulo, desde o primeiro caso da doença, identificado em 1980.

## MÉTODOS

### Desenho e Área do Estudo

Estudo descritivo ecológico na cidade de São Paulo, capital do estado de mesmo nome, com população estimada, em 2019, de 11,8 milhões de habitantes, sendo 47,6% homens. Sua área é de 1.521,11 km² e a densidade demográfica de 7.765,06 habitantes/km², de acordo com a Fundação Sistema Estadual de Análise de Dados^20^. As coordenadas geográficas do ponto central do município de São Paulo são: latitude de 23°32’51” Sul e longitude de 46°38’10” Oeste.

Para o planejamento de ações de saúde, o município está dividido em seis Coordenadorias Regionais de Saúde (CRS): Norte, Centro, Oeste, Sudeste, Leste e Sul. Possui 310 áreas de ponderação (AP), unidades de análise escolhidas para este estudo (Figura 1), formadas por agrupamentos mutuamente exclusivos de setores censitários do Instituto Brasileiro de Geografia e Estatística (IBGE)^21^.

**Figura 1:** (A) Mapa do Brasil, segundo unidades federadas, com destaque para o município de São Paulo. (B) Mapa do município de São Paulo, segundo Coordenadorias Regionais de Saúde (CRS) e áreas de ponderação.

Fonte: Mapas digitais da América do Sul e Coordenadorias Regionais de Saúde: CEInfo - Secretaria Municipal da Saúde de São Paulo (2018). Mapas digitais do Brasil, unidades federadas, município de São Paulo e áreas de ponderação: Instituto Brasileiro de Geografia e Estatística (2018).

### Fontes de Dados

Os dados para este trabalho provêm do banco de notificações de HIV/aids do Sistema de Informação de Agravos de Notificação (Sinan), da Coordenadoria de Vigilância em Saúde do município de São Paulo. A base cartográfica de logradouros do MSP foi obtida no Centro de Estudos da Metrópole (CEM)^22^. As malhas digitais e as populações das AP foram adquiridas no sítio eletrônico do IBGE^21^.

### População e Período do Estudo

Optou-se por descrever a trajetória da aids exclusivamente no sexo masculino, visto que, no município de São Paulo, dos 88.424 casos notificados entre 1980 e 2013, 72,2% eram homens^23^, proporção maior que as do estado de São Paulo (68,9%)^24^ e do Brasil (64,9%)^25^. Um total de 57.440 casos de Aids em homens residentes no MSP foram registrados no Sinan no período do estudo, de janeiro de 1980 a junho de 2012.

### Geocodificação dos Casos de Aids

A geocodificação dos casos de aids foi realizada conforme o endereço de moradia (logradouro, número, complemento, referência, bairro e CEP), para o relacionamento com o mapa digital de logradouros do MSP do CEM^22^ de 2014, disponível gratuitamente na internet, utilizando-se o *software* MapInfo Professional (versão 11.0), além de sistemas complementares baseados no *Google Earth*.

Foi necessário padronizar os endereços dos casos, por falta de uniformidade e incorreções, como também para compatibilizá-los à base digital de logradouros, antes de se obter as localizações geográficas. A qualidade da geocodificação foi verificada pela checagem de 10% dos endereços geocodificados, sorteados aleatoriamente, sobrepondo-se a camada dos pontos geocodificados à camada de logradouros do MSP, obtendo-se 96% de acurácia.

### Análises Estatísticas de Varredura

O presente trabalho buscou detectar aglomerados geográficos significativamente altos de aids em homens no espaço e no espaço-tempo. As análises estatísticas de varredura foram realizadas no programa SaTScan^TM^ (versão 9.4.2)^26^, partindo do pressuposto de que o número de casos em cada área de ponderação segue a distribuição de Poisson, segundo uma população de base sob risco conhecida^6^^,^^8^^,^^14^^,^^26^. O tamanho máximo de população para a janela de varredura foi de 5%. O valor de p dos aglomerados foi obtido por meio do teste de hipóteses de Monte Carlo, com 999 replicações (padrão do SaTScan^TM^)^26^. Para mais detalhes sobre a teoria por trás das estatísticas de varredura espacial e espaço-temporal, recomenda-se a consulta às referências de Kulldorff^8^^,^^26^^,^^27^.

A trajetória dos casos de aids foi analisada por três modalidades de estatísticas de varredura: (1) puramente espacial; (2) espaço-temporal; (3) de variação espacial nas tendências temporais. Estatísticas de varredura são usadas para detectar e avaliar aglomerados de casos em uma configuração puramente temporal, puramente espacial ou espaço-temporal. Isso é feito pela varredura gradual de uma janela ao longo do tempo e/ou do espaço, assinalando o número de observações observadas e esperadas no interior da janela em cada localidade^26^.

Os mapas com os aglomerados significativos (p < 0,05) e respectivos riscos relativos (análises de varredura espacial e espaço-temporal), e tendências temporais internas e externas (análise de variação espacial nas tendências temporais) foram elaborados no aplicativo ArcGIS (versão 10.1).

Para cada modalidade, foram avaliados três conjuntos de dados: (a) todos os casos de aids em homens com 13 anos de idade ou mais – faixa etária considerada pela vigilância como “aids em adultos”^28^; (b) homens com 50 anos ou mais, devido à importância crescente de casos de aids em idades mais avançadas, observada desde o final da década de 1990^2^^,^^3^; (c) óbitos por aids com 13 anos de idade ou mais. Para cada análise, foi necessária a criação, requerida pelo SaTScan^TM^, de três arquivos^26^:

1. Arquivo de casos: contém o número de casos em cada AP. Para as análises espaço-temporais, o referencial de tempo foi a data de diagnóstico de aids, no formato ano/mês. A idade foi incluída como uma covariável, estratificada nas seguintes faixas etárias: 10–19; 20–29, 30–39, 40–49, 50–59, 60–69 e 70 ou mais.
2. Arquivo de população: compreende um tamanho de população sob risco para cada combinação de localidade (AP), tempo (em anos) e covariável. As populações segundo as faixas etárias foram especificadas para cada AP nos anos censitários (1980, 1991, 2000 e 2010). Para os anos intercensitários, o SaTScan^TM^ realizou uma interpolação linear baseada nas populações dos censos imediatamente anterior e posterior^26^.
3. Arquivo de coordenadas espaciais: pares de coordenadas de latitude e longitude dos centroides das AP, utilizadas como um *proxy* para a localização dos participantes do estudo^6^^,^^11^.

### Aspectos Éticos

A utilização dos dados para fins deste trabalho foi aprovada pelo Comitê de Ética em Pesquisa da Faculdade de Saúde Pública da Universidade de São Paulo (parecer consubstanciado nº 261.202, de 19 de abril de 2013). Para quaisquer circunstâncias, foram consideradas as normas e diretrizes que regulamentam as pesquisas envolvendo seres humanos (resolução nº 466, de 12 de dezembro de 2012, do Conselho Nacional de Saúde).

## RESULTADOS

Do total de 57.440 casos de aids em homens, 53.825 foram geolocalizados em várias etapas, atingindo uma taxa final de geocodificação de 93,7%. Foram excluídos 53 casos com idade ignorada. Assim, para cada conjunto de dados, foram analisados: (a) 53.772 casos com 13 anos de idade ou mais; (b) 5.272 casos com 50 anos ou mais; (c) 28.981 óbitos por aids.

No que se refere à análise puramente espacial do total de casos, foram identificados 12 aglomerados espaciais de alto risco estatisticamente significativos (p < 0,05). Em todo o período analisado, a epidemia se concentrou no Centro do MSP, com o aglomerado principal com risco relativo (RR) de 4,90 (Figura 2A). Os dois aglomerados secundários de maior significância estatística foram localizados, respectivamente, ao Norte e a Sudeste do município, próximos ao aglomerado principal (Figura 2A).

**Figura 2:** Mapas dos aglomerados puramente espaciais com altas taxas de casos de aids em homens, segundo área de ponderação, Coordenadoria Regional de Saúde (CRS) e risco relativo (RR): (A) total de casos com 13 anos de idade ou mais; (B) total de casos com 50 anos de idade ou mais; (C) óbitos com 13 anos de idade ou mais. Município de São Paulo, 1980 a junho de 2012.

Fonte: Casos de aids: Sistema de Informação de Agravos de Notificação – Centro de Controle de Doenças da Coordenadoria de Vigilância em Saúde da Secretaria Municipal da Saúde de São Paulo. População: Datasus/Instituto Brasileiro de Geografia e Estatística (IBGE). Áreas de ponderação: censo do IBGE (2010). CRS: CEInfo – Secretaria Municipal da Saúde de São Paulo.

Na análise dos casos com 50 anos ou mais, identificaram-se três aglomerados significativos. O principal, com RR de 5,90 (Figura 2B) ocupou exatamente a mesma área central do aglomerado principal do conjunto de todos os casos (Figura 2A).

Na análise de varredura espacial dos óbitos, foram encontrados oito aglomerados significativos. O principal, com RR de 4,44 (Figura 2C) permaneceu no Centro da cidade, envolvendo as mesmas AP anteriores, além de duas AP a Oeste (Figura 2C). Levando-se em conta puramente o espaço, a epidemia de aids nos homens apresentou uma importante concentração espacial no Centro do município e em áreas contíguas das regiões Norte, Sudeste e Oeste, independentemente da faixa etária e da evolução para o óbito.

Por meio da estatística de varredura espaço-temporal do total de casos, foram identificados 16 aglomerados significativos. O principal, com RR de 7,20 (Figura 3A) ocorreu entre outubro de 1987 e março de 2004, no Centro da cidade. Os outros 15 aglomerados secundários, concentrados principalmente no período de 1993 a 1999, distribuíram-se nas demais regiões do município, inclusive na CRS Sul, que contou com três aglomerados (Figura 3A).

**Figura 3:** Mapas dos aglomerados espaço-temporais com altas taxas de casos de aids em homens, segundo área de ponderação, Coordenadoria Regional de Saúde (CRS), período do aglomerado e risco relativo (RR): (A) total de casos com 13 anos de idade ou mais; (B) total de casos com 50 anos de idade ou mais; (C) óbitos com 13 anos de idade ou mais. Município de São Paulo, 1980 a junho de 2012.

Fonte: Casos de aids: Sistema de Informação de Agravos de Notificação – Centro de Controle de Doenças da Coordenadoria de Vigilância em Saúde da Secretaria Municipal da Saúde de São Paulo. População: Datasus/Instituto Brasileiro de Geografia e Estatística (IBGE). Áreas de ponderação: censo do IBGE (2010). CRS: CEInfo – Secretaria Municipal da Saúde de São Paulo.

Na faixa etária com 50 anos ou mais, foram detectados sete aglomerados significativos. O principal, com RR de 8,61 (Figura 3B), situou-se na mesma área central dos aglomerados principais nas análises puramente espaciais, e outros cinco aglomerados secundários ocorreram no Sudeste, Oeste e Norte, “tocando” o principal. Os períodos mais críticos ocorreram entre 1992 e 1999; contudo, destaca-se um aglomerado com RR de 1,73 que perdurou até 2012, ano mais recente do estudo (Figura 3B).

Na análise espaço-temporal dos óbitos, foram encontrados 15 aglomerados significativos. O principal, com RR de 8,36 (Figura 3C) permaneceu na região central, agora mais ampliado, entre fevereiro de 1987 e setembro de 1999. Destaca-se a expansão de aglomerados secundários na CRS Leste, além de três aglomerados localizados exclusivamente na região Sul. O período mais crítico ocorreu entre 1990 e 1996 (Figura 3C). Na análise de varredura que levou em conta simultaneamente o espaço e o tempo, foram encontrados diversos aglomerados espalhados por todas as regiões do município.

Na análise de variação espacial nas tendências temporais, foram encontrados nove aglomerados significativos no conjunto do total de casos. Apresentam-se os valores das tendências temporais internas (INT) e externas (EXT) dos aglomerados. Com incremento anual interno de 4,52%, o aglomerado mais significativo ocorreu na região Sul do MSP (Figura 4A).

**Figura 4:** Mapas dos aglomerados de variação espacial nas tendências temporais com altas tendências de casos de aids em homens, segundo área de ponderação, Coordenadoria Regional de Saúde (CRS), tendência temporal de incremento anual (%) dentro (INT) e fora (EXT) do aglomerado: (A) total de casos com 13 anos de idade ou mais; (B) total de casos com 50 anos de idade ou mais; (C) óbitos com 13 anos de idade ou mais. Município de São Paulo, 1980 a junho de 2012.

Fonte: Casos de aids: Sistema de Informação de Agravos de Notificação – Centro de Controle de Doenças da Coordenadoria de Vigilância em Saúde da Secretaria Municipal da Saúde de São Paulo. População: Datasus/Instituto Brasileiro de Geografia e Estatística (IBGE). Áreas de ponderação: censo do IBGE (2010). CRS: CEInfo – Secretaria Municipal da Saúde de São Paulo.

Na faixa etária de 50 anos ou mais, foram detectados apenas três aglomerados significativos. O principal, com incremento anual interno de 7,58%, também foi localizado na CRS Sul, próximo à fronteira com a região Oeste (Figura 4B).

No subconjunto dos óbitos, foram detectados sete aglomerados significativos. O principal, com incremento anual interno de 1,23% (Figura 4C), envolveu as mesmas AP e situou-se na mesma região do aglomerado principal do conjunto de todos os casos (Figura 4A).

Independentemente da faixa etária e da evolução dos casos de aids em homens, existem aglomerados espaciais, sobretudo nas regiões mais periféricas do MSP, que apresentaram maior incremento percentual anual das taxas da doença dentro dos aglomerados, em comparação às áreas externas a eles, o que denota uma tendência de “periferização” da epidemia nos homens neste município.

Na Tabela, resumem-se as principais características dos aglomerados principais e secundários estatisticamente significativos de casos de aids em homens nos três conjuntos de dados analisados, identificados nas análises de varredura puramente espacial, espaço-temporal e de variação espacial nas tendências temporais.

**Tabela:** Características dos aglomerados principais e secundários estatisticamente significativos (p < 0,05) de altas taxas de aids em homens, identificados nas análises estatísticas de varredura puramente espacial, espaço-temporal e de variação espacial nas tendências temporais, segundo diferentes conjuntos de dados analisados. Município de São Paulo, 1980 a junho de 2012.

Conjunto de dados	Análise de varredura puramente espacial								
	Tipo de aglomerado	n aglomerados	n localidades	n casos observados	n casos esperados	Obs/Esp^a^	Risco relativo		p
Homens - 13 anos de idade ou mais	Principal	1	15	9.164	2.163,1	4,24	4,90		< 0,0001
	Secundários^b^	11	61	14.175	10.358,3	1,21–1,64	1,22–1,66		< 0,0021
Homens - 50 anos de idade ou mais	Principal	1	15	1.016	204,9	4,96	5,90		< 0,0001
	Secundários^b^	2	9	220	145,7	1,40–2,19	1,42–2,20		< 0,013
Óbitos em homens	Principal	1	16	4.603	1.181,1	3,90	4,44		< 0,0001
	Secundários^b^	7	69	8.667	6.276,7	1,23–1,81	1,23–1,88		< 0,036

Conjunto de dados	Análise de varredura espaço-temporal								
	Tipo de aglomerado	n aglomerados	n localidades	n casos observados	n casos esperados	Obs/Esp^a^	Risco relativo	Período^c^	p
Homens - 13 anos de idade ou mais	Principal	1	15	6.880	1.074,1	6,40	7,20	10/1987-03/2004	< 0,0001
	Secundários^b^	15	163	15.645	7.916,9	1,44–3,24	1,44–3,25	09/1987-09/2003	< 0,026
Homens - 50 anos de idade ou mais	Principal	1	15	729	96,5	7,56	8,61	08/1987-01/2004	< 0,0001
	Secundários^b^	6	55	715	367,2	1,70–3,99	1,72–4,01	02/1989-04/2012	< 0,05
Óbitos em homens	Principal	1	19	3.904	529,8	7,37	8,36	02/1987-09/1999	< 0,0001
	Secundários^b^	14	156	9.991	3.653,7	1,66–3,85	1,67–3,85	01/1988 –11/2000	< 0,0001

Conjunto de dados	Análise de variação espacial nas tendências temporais								
	Tipo de aglomerado	n aglomerados	n localidades	n casos observados	n casos esperados	Obs/Esp^a^	Risco relativo	Tendência dentro do aglomerado	p
Homens - 13 anos de idade ou mais	Principal	1	14	1.571	2.648,3	0,59	0,58	4,52	0,001
	Secundários^b^	8	95	10.580	17.144,8	0,46–0,79	0,45–0,78	3,32–6,13	< 0,0041
Homens - 50 anos de idade ou mais	Principal	1	17	179	259,7	0,69	0,68	7,58	0,003
	Secundários^b^	2	34	337	485,0	0,66–0,73	0,65–0,72	7,13–7,35	< 0,03
Óbitos em homens	Principal	1	14	790	1.424,6	0,55	0,54	1,23	0,001
	Secundários^b^	6	80	4.488	7.731,8	0,40–0,78	0,39–0,77	0,25–1,27	< 0,011

a Obs/Esp: relação entre casos observados e esperados.

b As características dos aglomerados secundários foram resumidas pela soma do número de localidades, casos observados e casos esperados de todos os aglomerados. A razão entre observado e esperado, os riscos relativos e as tendências internas dos aglomerados são apresentados segundo os valores mínimos e máximos.

c O período relatado para os aglomerados secundários refere-se à primeira data (mês/ano) do aglomerado espaço-temporal mais precocemente identificado, e à data (mês/ano) final do aglomerado que teve o término mais tardio.

Fonte: Arquivo de informações de saída do SaTScan^TM^ (versão 9.4.2, 2015).

## DISCUSSÃO

Este estudo procurou identificar aglomerados espaciais e espaço-temporais de aids em homens com 13 anos de idade ou mais no município de São Paulo, desde o aparecimento do primeiro caso, em 1980, até junho de 2012. Diversos métodos analisam a aglomeração global e local, com diferenças de desempenho em termos de poder e precisão^4^^–^^6^^,^^9^^–^^19^. No presente, optou-se pelo método de varredura, tendo em vista o objetivo de identificar localidades com alta concentração de aids em homens.

A progressão do HIV nas populações não ocorre de modo aleatório e uniforme, mas sim em função de comportamentos sociais íntimos e complexos entre duas ou mais pessoas^11^^,^^14^. Apesar do papel determinante de tais comportamentos interativos na transmissão da doença, a abordagem baseada no indivíduo não é suficiente para uma avaliação plena do papel desempenhado pelas interações dos grupos, que redundam na disseminação do HIV^14^. Para que ocorra a transmissão da doença, muitos pequenos grupos devem estar vinculados uns aos outros, ou ainda a componentes maiores, por conexões não identificadas. A informação desses pequenos componentes pode ser difícil de analisar, haja vista a falta de conhecimento relativo a outros componentes aos quais eles podem estar vinculados^14^.

O uso de ferramentas de análise espacial permite identificar diferenciais no comportamento do HIV/aids nas populações e nos espaços que ocupam. Tais técnicas contribuem também para a identificação de localidades onde há maior risco de adoecer ou morrer em função da doença, o que pode auxiliar a selecionar áreas para políticas e intervenções específicas por parte dos órgãos competentes^29^.

A análise de varredura puramente espacial evidenciou que a aids se concentrou no Centro do município de São Paulo, independentemente da faixa etária e da evolução para o óbito. O Boletim Epidemiológico de Aids, HIV e DST do MSP de 2014^30^ menciona uma epidemia concentrada na região central, apesar da importante redução da taxa de incidência observada nessa área entre 2005 e 2013. Um estudo realizado em 2015 identificou uma prevalência de HIV de 15,4% entre homens que fazem sexo com homens (HSH) no Centro do MSP, com desconhecimento de mais da metade da população estudada quanto à sua soropositividade, levantando questionamentos sobre uma “nova onda” da epidemia da doença^31^.

O risco relativo do aglomerado de homens com 50 anos ou mais de idade foi o maior dentre todos aqueles observados nas análises puramente espaciais. Entre 1996 e 2005, a taxa de incidência de aids entre homens de 50 a 59 anos passou de 18,2 para 29,8 casos/100.000 homens. Nos maiores de 60 anos, no mesmo período, também houve aumento da taxa, de 5,9 para 8,8. Até 2010, tal aumento se manteve nas faixas etárias acima dos 50 anos^2^^,^^3^.

O “envelhecimento” da população com aids tem sido destacado como uma tendência da epidemia no Brasil, e os idosos raramente são alvo de intervenções para a redução do contágio^2^^,^^15^^,^^32^. Considerando a história natural da doença, é válido supor que a infecção por HIV nesse grupo tenha ocorrido pelo menos 10 anos antes da manifestação da aids. Infere-se que os idosos iniciaram sua vida sexual quando o HIV/aids ainda não existia, de modo que são menos propensos a usar preservativos, apresentando maior exposição ao vírus. Alguns autores consideram ainda que o aumento da incidência nos homens mais idosos pode ser um reflexo da utilização de medicamentos contra a impotência, que favorece a vida sexual ativa na terceira idade^1^^,^^32^.

Segundo dados recentes do Ministério da Saúde, na última década observou-se um incremento da taxa de detecção de aids nos homens de 15 a 29 anos e com 55 anos ou mais, com destaque para os jovens de 15 a 24 anos^25^. A Pesquisa de Conhecimentos, Atitudes e Práticas na População Brasileira (PCAP)^33^, realizada em 2013 com indivíduos de 15 a 64 anos, apontou que, dentre as razões para a “segunda onda” de infecção entre gays e HSH, estão o baixo índice de testagem (86,1% das pessoas do sexo masculino nunca haviam se testado para o HIV na vida) e a ocorrência de maior número de parcerias casuais entre jovens, além de maior percentual de relações sexuais com pessoas conhecidas por meio da internet^33^.

Na análise de varredura espaço-temporal, foi observado um número expressivo de aglomerados, denotando que todas as regiões do município foram acometidas em algum momento, exceto a Sul. O aglomerado principal se manteve entre outubro de 1987 e março de 2004, no Centro da cidade, incluindo o ano de 1992, que apresentou a maior taxa de incidência de aids em homens adultos (88,1 casos/100.000 homens)^29^.

A semelhança do subconjunto de óbitos com aquele que incluiu todos os casos pode ser explicada pela alta letalidade da doença nos homens (54,7%), sobretudo entre 1990 e 1996^29^. A partir desse ano, avanços no tratamento da doença, incluindo a distribuição gratuita da terapia antirretroviral de alta potência (TARV) pelo Ministério da Saúde, traduziram-se em expressiva melhora na sobrevida dos pacientes, com queda da mortalidade em cerca de 50% e redução das infecções oportunistas^16^.

O pico da mortalidade por aids em homens no município de São Paulo ocorreu em 1994 (52,5/100.000 homens)^23^^,^^30^; a partir de então, medidas voltadas à assistência redundaram em uma queda expressiva da taxa bruta de mortalidade (TBM), que em 2012 foi de 10,4/100.000 homens^23^. No mesmo ano, no estado de São Paulo, essa taxa foi de 9,1/100.000 homens^24^, e no Brasil, de 8,3/100.000 homens^25^.

Uma análise realizada com as declarações de óbito (DO) do Sistema de Informações sobre Mortalidade (SIM) com menção de HIV/aids em qualquer campo de preenchimento das causas de óbito mostrou que no Brasil, entre 2000 e 2007, ocorreu um importante aumento no percentual de óbitos de pessoas infectadas pelo vírus por causas básicas não relacionadas ao HIV/aids, passando de 2,5% para 7,0%. Esse fato indica que outros agravos (neoplasias malignas, doenças cardiovasculares, doenças do aparelho digestivo e respiratório, doenças metabólicas e endocrinológicas, hepatites virais e causas externas) têm impactado de forma crescente na sobrevida das pessoas com HIV/aids^2^.

Fatores como o diagnóstico tardio, coinfecções, a falta de acesso aos recursos de saúde, entre outros, atingem as pessoas vivendo com HIV/aids de forma mais intensa em algumas localidades do que em outras^34^. O diagnóstico precoce do HIV, de modo a garantir o tratamento oportuno, a adesão ao tratamento, o fortalecimento da rede de genotipagem e a inclusão de novos medicamentos são estratégias fundamentais para a redução da mortalidade por aids e para a melhoria da qualidade de vida das pessoas vivendo com HIV/aids^29^^,^^34^.

Nesse estudo da trajetória da aids no município de São Paulo, evidenciou-se uma tendência de “periferização” da doença nos homens, visto que áreas mais periféricas apresentaram, ao longo do tempo, maior incremento percentual das taxas dentro dos aglomerados do que as áreas externas a eles. Destaca-se que um aglomerado estatisticamente significativo nessa modalidade de análise não implica, necessariamente, que a taxa global da doença seja maior ou menor nele, visto que a estatística de varredura da variação espacial nas tendências temporais não está à procura de aglomerados de alto ou baixo risco, mas sim busca aglomerados com uma tendência interna maior ou menor do que a tendência fora deles^26^.

Os estratos mais pobres são os mais afetados pela aids, em função de diversos aspectos relacionados à vulnerabilidade, como falta de recursos para a prevenção do HIV; dificuldade de acesso a serviços de saúde; situações de constrangimento, pressão e ameaças; precariedade de moradia, alimentação e emprego; baixa escolaridade e difícil acesso à informação^35^. A presença da epidemia de aids em contextos de vulnerabilidade social agrava as condições já precárias desses estratos, pois acumulam-se condições de vida indesejáveis^29^.

Em 1994, já se sinalizava a “pauperização” da aids, que acometia principalmente os estratos sociais mais pobres e moradores da periferia^36^. O processo de “pauperização” representou uma das tendências da epidemia da doença no Brasil, que atingiu de forma crescente pessoas com menor grau de escolaridade e usuários de drogas injetáveis^4^^,^^5^^,^^16^^,^^36^. A exclusão territorial se relaciona à exclusão social, refletindo os processos de urbanização e de segregação urbana^37^. O centro e a periferia do MSP representam um exemplo dessa desigualdade.

Este trabalho apresenta algumas limitações, dentre as quais destaca-se terem sido estudados somente os casos de aids, não incluindo os de HIV assintomáticos, pois apenas a aids era agravo de notificação obrigatória no período do estudo. O período de latência prolongado da aids dificulta a caracterização do local onde os indivíduos adquiriram o HIV, pois quando são notificados com infecções definidoras de aids, um longo tempo já se passou desde o momento da infecção. Outra limitação diz respeito ao endereço de moradia dos pacientes informado nas fichas de notificação e investigação de aids, que pode não representar o verdadeiro local de residência, pois endereços imprecisos são fornecidos com o objetivo de se conseguir acesso à rede de assistência fora da região de moradia.

Considera-se, ainda, a possibilidade de sub-registros da doença nos sistemas de vigilância e de mortalidade, principalmente nas primeiras décadas da epidemia, em função de mudanças nas versões da Classificação Internacional de Doenças (no CID-9, vigente até 1995, não havia uma categoria específica para aids), além do estigma relacionado à doença, que pode ter colaborado para a não menção dessa causa nas declarações de óbito. Além disso, as técnicas de varredura não possibilitam verificar associações entre exposição e desfecho, revelando apenas as localidades com maior risco ou proteção nas taxas da doença.

Como ponto forte do estudo, destaca-se a expressiva proporção de casos de aids geocodificados (93,7%). Além disso, o delineamento ecológico permitiu evidenciar efeitos não detectados no plano individual, e a opção por técnicas de varredura espacial, espaço-temporal e de variação espacial nas tendências temporais possibilitou a detecção de aglomerados geográficos de alto risco de aids em homens.

Em conclusão, a epidemia de aids nos homens se concentrou na região central do município de São Paulo e em áreas contíguas das regiões Norte, Sudeste e Oeste, independentemente da faixa etária analisada e da evolução para o óbito. Ao longo do tempo, a doença alcançou homens em todas as regiões do município, com tendência de “periferização”. O estudo auxiliou a revelar áreas prioritárias no município de São Paulo, tanto para ações programáticas no âmbito do HIV/aids como para nortear outros estudos.
